# Supplementary material for: COZOID: contact zone identifier for visual analysis of protein-protein interactions
Source: BMC Bioinformatics. 2018 Apr 6;19:125. doi: 10.1186/s12859-018-2113-6 (PMC5889581; doi:10.1186/s12859-018-2113-6)
Supplement: Supplementary file 2 — Software build. Executable binary file of the software tool. (ZIP 118,784 kb) [file 12859_2018_2113_MOESM2_ESM.zip › cozoid/platform/docs/testng-6.5.1-javadoc.zip!/org/testng/annotations/IParameterizable.html]

IParameterizable (TestNG)


---


|  |  |  |
| --- | --- | --- |
| **Overview** \| **Package** \| **Class** \| **Tree** \| **Deprecated** \| **Index** \| **Help** | |  |
| **PREV CLASS**   **NEXT CLASS** | **FRAMES**    **NO FRAMES**     **All Classes** |  |
| SUMMARY: NESTED \| FIELD \| CONSTR \| METHOD | DETAIL: FIELD \| CONSTR \| METHOD |  |


---


## org.testng.annotations Interface IParameterizable

**All Superinterfaces:**: IAnnotation

**All Known Subinterfaces:**: IConfigurationAnnotation, IFactoryAnnotation, ITestAnnotation, ITestOrConfiguration

---

``` public interface IParameterizable extends IAnnotation ```

Parent interface for annotations that can receive parameters.
Created on Dec 20, 2005

**Author:**
:   Cedric Beust

---

| **Method Summary** | |
| --- | --- |
| `boolean` | `getEnabled()`             Whether this annotation is enabled. |
| `java.lang.String[]` | `getParameters()`             **Deprecated.** *Use @Parameters* |
| `void` | `setEnabled(boolean enabled)` |

**Method Detail**

### getParameters

```
@Deprecated
java.lang.String[] getParameters()
```

:   **Deprecated.** *Use @Parameters*

    :   The list of variables used to fill the parameters of this method.
        These variables must be defined in the property file.

---


### getEnabled

```
boolean getEnabled()
```

:   Whether this annotation is enabled.

---


### setEnabled

```
void setEnabled(boolean enabled)
```


---


|  |  |  |
| --- | --- | --- |
| **Overview** \| **Package** \| **Class** \| **Tree** \| **Deprecated** \| **Index** \| **Help** | |  |
| **PREV CLASS**   **NEXT CLASS** | **FRAMES**    **NO FRAMES**     **All Classes** |  |
| SUMMARY: NESTED \| FIELD \| CONSTR \| METHOD | DETAIL: FIELD \| CONSTR \| METHOD |  |


---
